# Supplementary material for: A spatially resolved atlas of the human lung characterizes a gland-associated immune niche
Source: Nat Genet. 2022 Dec 21;55(1):66–77. doi: 10.1038/s41588-022-01243-4 (PMC9839452; doi:10.1038/s41588-022-01243-4)
Supplement: Supplementary file 1 — Supplementary table, file and figure legends; Supplementary Figures 1–4 [file 41588_2022_1243_MOESM1_ESM.pdf]

nature genetics

Article https://doi.org/10.1038/s41588-022-01243-4

A spatially resolved atlas of the human lung characterizes a gland-associated immune niche

In the format provided by the authors and unedited

Supplementary legends

Supplementary Table 1: Donor metadata. Information on age range, sex, BMI range, smoking status, years smoking and ethnic origin of donors.

Supplementary Table 2: Sample information for single cell and single-nuclei RNA-seq runs.
Sample ID-s for scRNAseq and corresponding location, spatial code, material of cells/nuclei, protocol, enrichment and dissociation notes, Donor ID-s with age, BMI, sex and smoking history if available, 10x version, Gene expression sequencing run ID and corresponding BCR and TCR sample ID-s for single cell RNA-seq samples. For snRNAseq, sample ID-s and corresponding information for snRNAseq runs regarding pooling, donors, location, protocol and 10x version.

Supplementary Table 3: Sample information for Visium Spatial Transcriptomic sequencing runs. Sample and image ID-s, location and donor information, permeabilisation time and Visium slide ID for Visium spatial transcriptomics samples.

Supplementary Table 4: Cell type annotation comparisons.
Comparison of the finest level of annotations in the current study with the finest level of annotations from the Integrated HLCA[12], the previous most recent healthy lung cell atlas[6] , the LungMAP integrated atlas[13] and the corresponding 'CellCards' from the LungMAP literature review[13].

Supplementary Table 5: Manual annotation of micro-anatomical tissue environments on Visium spatial transcriptomics. Manual annotation indicates presence (y) of various tissue regions on every Visium spatial transcriptomics sample. The annotations on the tissue can be seen as categories on the accompanying loupe files stored in DCP and in the online portal.

Supplementary Table 6: Antibody information for IBEX and IHC staining. Staining cycle, antibody protein names, clone, conjugate, Vendor, catalogue number and dilution used.

Supplementary Table 7: Proportions and numbers of cells from scRNA-seq and snRNA-seq data by Location, Material and Donor. Cell type groups "Celltypes master high" as shown in Figure 1e.

Supplementary Table 8: Output of fGWAS analysis for Lung function (FEV1/FVC) study (Figure 2f). Shown are the Log odds ratio (log OR), upper and lower confidence intervals (CI), p-value and FDR for each cell type. LogORs have been directly obtained as the beta values from fGWAS analysis. p-values were calculated using a two-sided Wald test on the coefficients, testing difference from 0. Benjamini-Hochberg multiple testing correction was performed over the 76 cell types.

Supplementary Table 9: Overview of donor inclusion across study. Use of donor material for a particular experiment (scRNAseq, snRNAseq, scVDJ-seq, fresh Visium, FFPE Visium, specific RNAscope/IHC validation experiments).

Supplementary Table 10: Significantly upregulated genes from comparison of peribronchial fibroblasts in COPD patients and controls from Adams et al. 2020 Sci Adv. scRNAseq. All 112 upregulated genes are displayed, with a subset of these which are relevant for COPD highlighted in Extended data 4c.

Supplementary Figure 1: Overview of Spatial Transcriptomics slides used in the study. H&E staining as well as the number of UMI counts per spot are visualised for each section. Full details for each sample is in Supplementary Table 3. Scale bar: 2mm.

Supplementary Figure 2: Donors' contribution to cell types. All identified cell types are seen across multiple donors (at least 5) and shown graphically by proportions of cell types identified per donor in (a) epithelial cells, (b) stroma and (c) immune cells. The effect of each donor contributing to the cell type proportion was calculated using cell type proportion analysis with a Poisson linear mixed model accounting for location, donor, sequencing material and dissociation protocol (d-f). The effect of donor as fold change and Local True Sign Rate (LTSR) score are shown for master cell types as additional data to Figure 1 (d), Fibroblasts as additional data to Figure 2 (e) and vasculature & SM as additional data to Figure 3 (e). Cell type numbers shown in Supplementary Table 7 and in online portal.

Supplementary Figure 3: Gene expression changes from veins to arteries for pulmonary and systemic circulation vascular endothelial and perivascular cell types. Visualisation of marker genes for cell types in these tissue microenvironments along this gradient: (a) pulmonary endothelial axis: arterial to CapA to venous cell types, (b) systemic endothelial axis: arterial to capillary to venous, (c) systemic smooth muscle: smooth muscle to pericyte to IR-Ven-Peri, (d) pulmonary smooth muscle: smooth muscle (SM) to pericyte.

Supplementary Figure 4: Unsupervised cell type co-localisation analysis on spatial transcriptomics data. Cell2location non-negative factorisation (NMF) analysis was used to map cell types on tissue sections and compared to the manual annotation. Cell abundance (manual annotations) and proportion per factor (factor analysis) is shown in the dotplots by color and dot size. Cell type enrichment in the (a) manually annotated regions of frozen Visium, and in the regions obtained by the clustering of spots into 8 (b), 11 (c) and 21 (d) unsupervised regions (factors). Different microenvironments are highlighted with boxes of different colours. The consistencies between manual annotation and NMF microenvironments are highlighted by solid lines. Additional microenvironments revealed by NMF analysis is shown with dotted and dashed lines. (e) Mapping of epithelial factors from b (cyan and orange, factors 0, 1, 2, 7 and 9 in b) on a representative bronchi section with corresponding H&E. Scale shows mean UMI for each factor in each Visium voxel. Scale bar: 1mm. Donors used for replicas are shown in Supplementary Table 9.

Supplementary Note 1. Cell type marker genes for all the described cell types.

Supplementary Figure 1

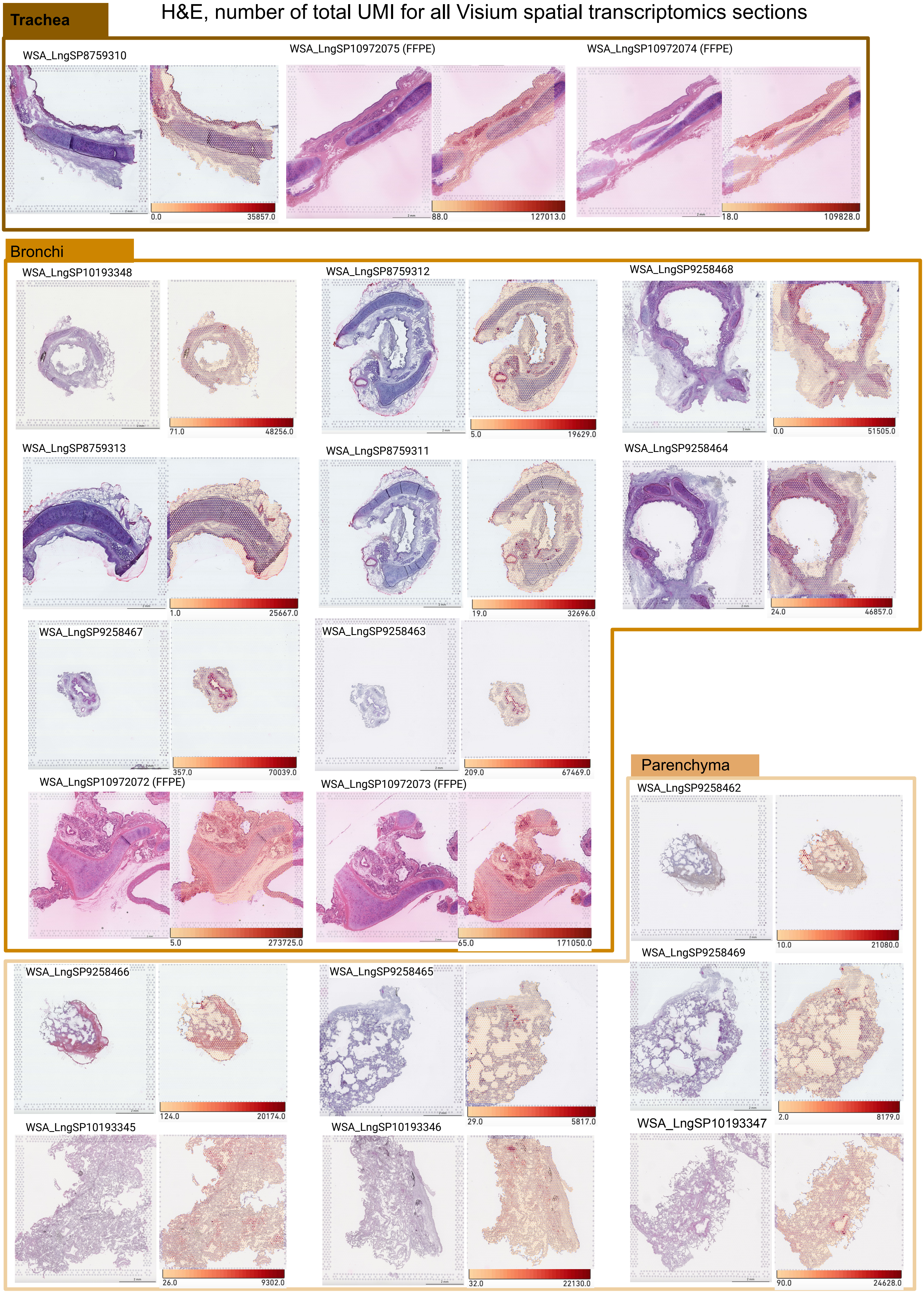

Supplementary Figure 2

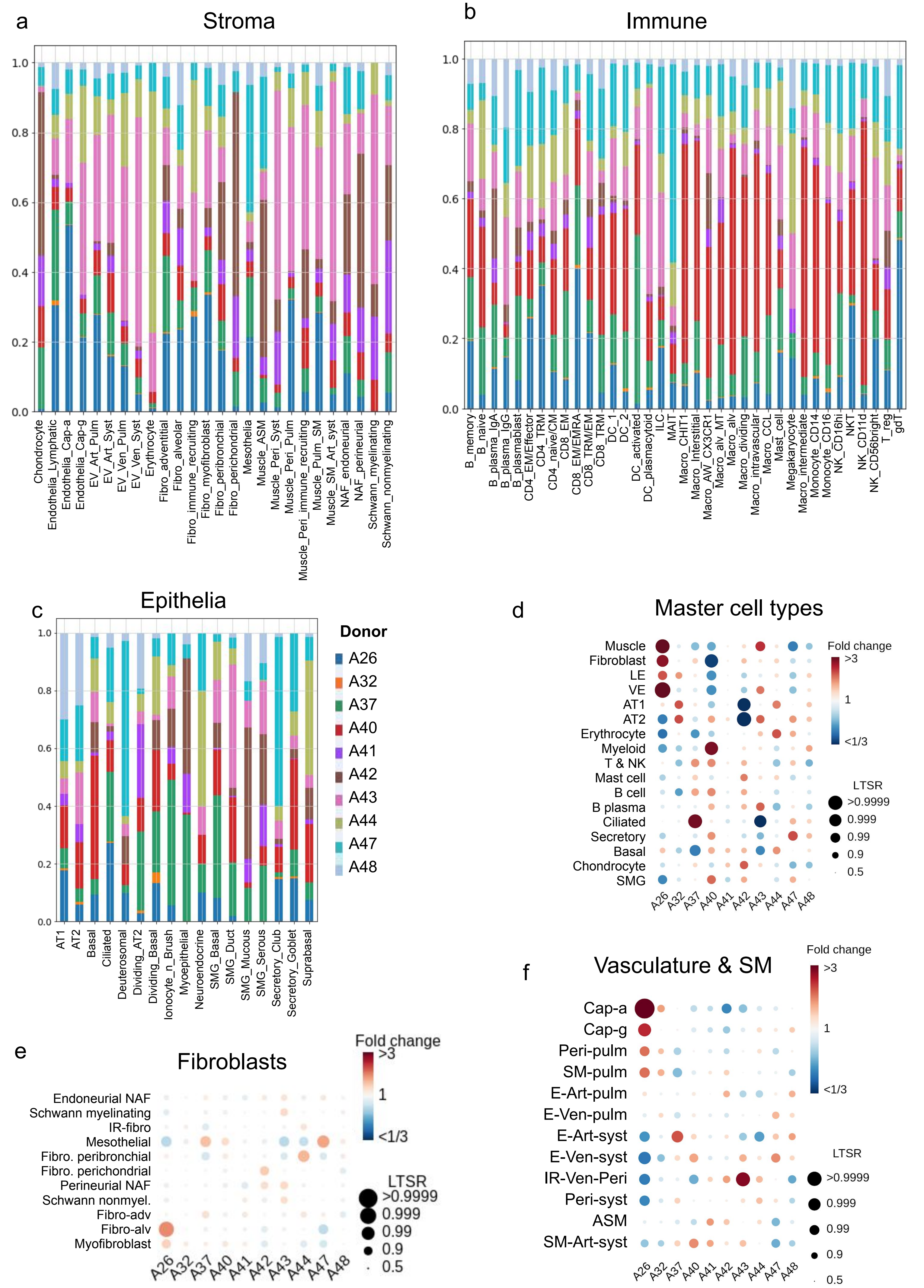

Supplementary Figure 3

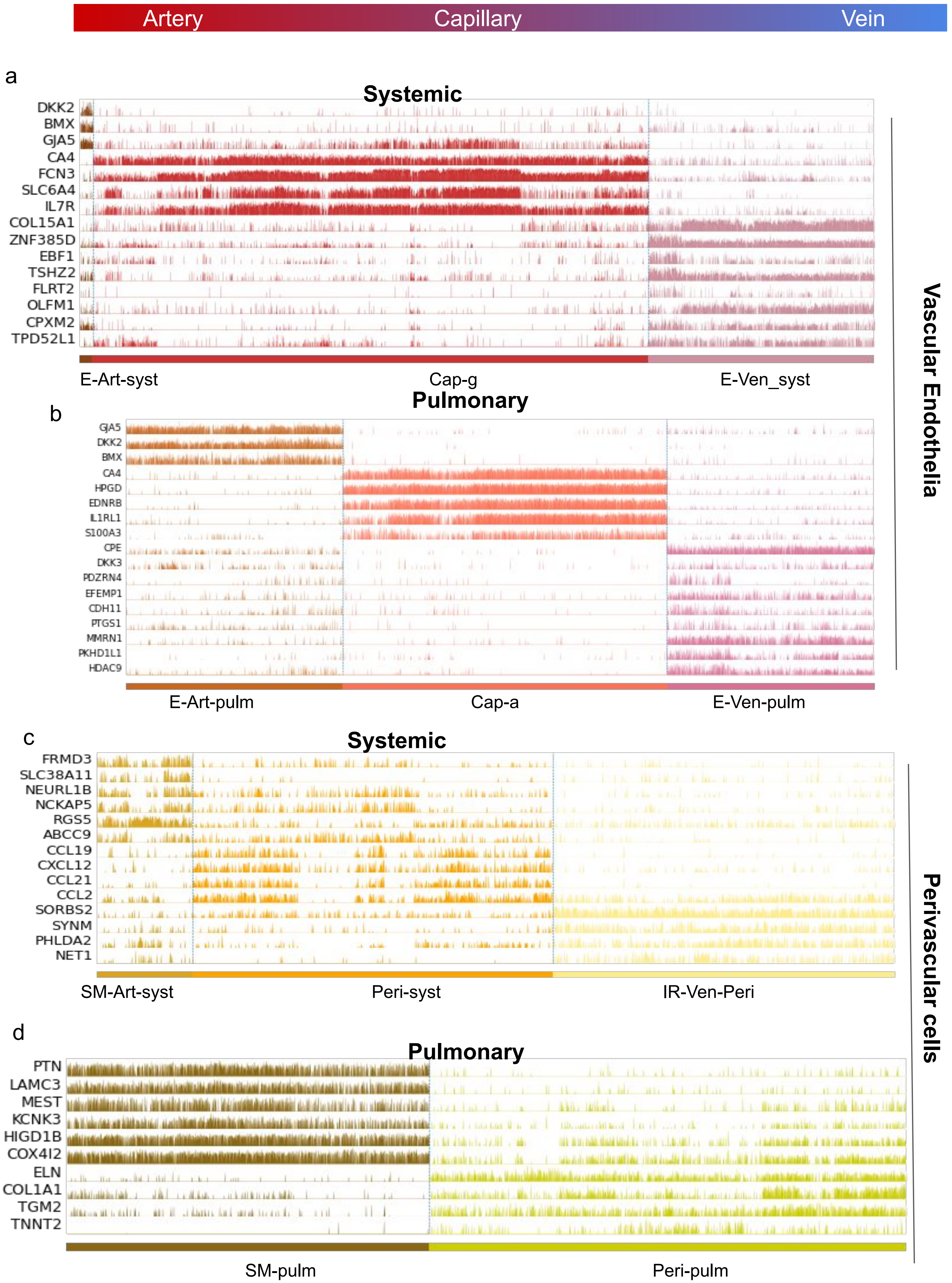

Supplementary Figure 4

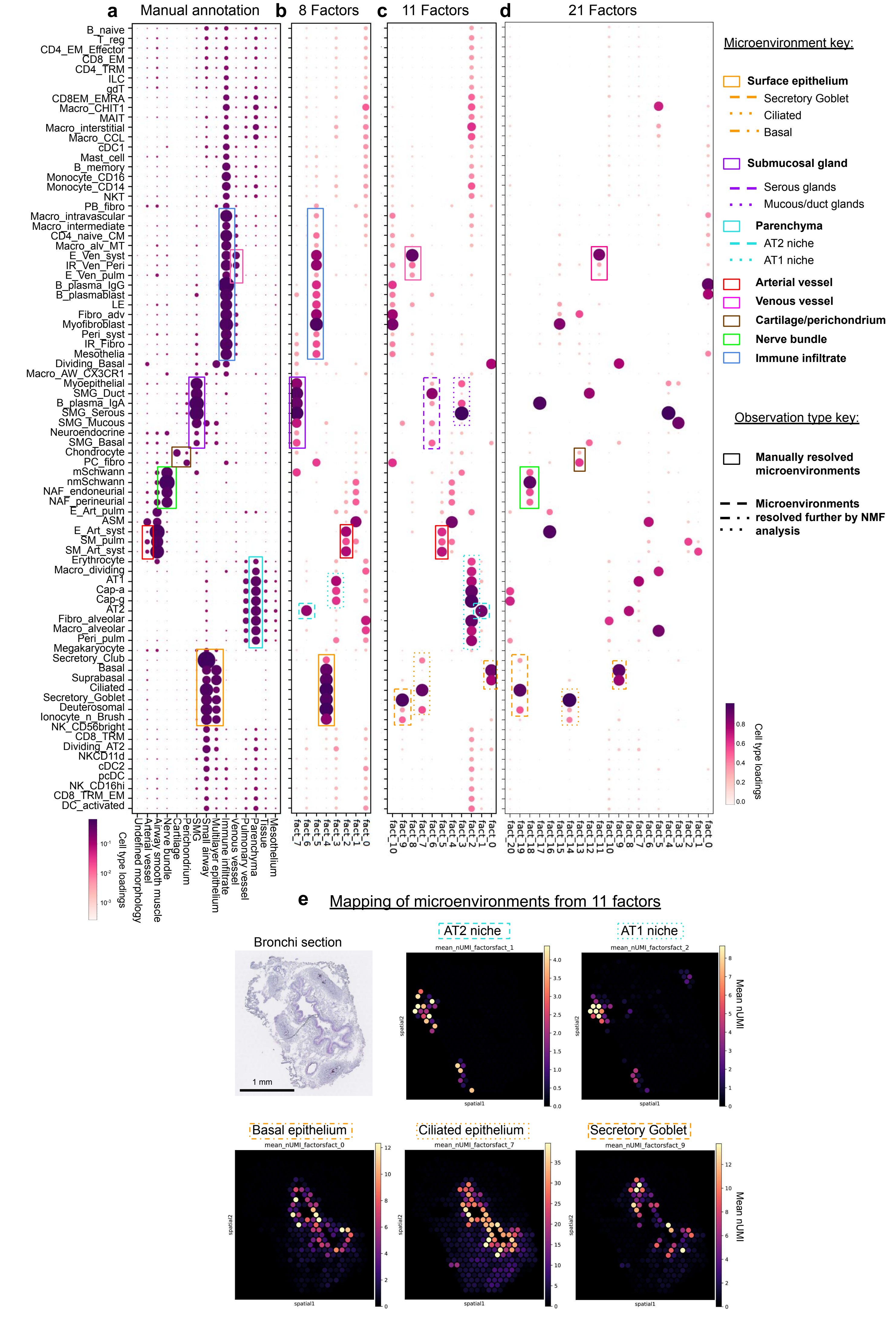

Supplementary Note 1: Cell type markers

##Epithelial markers

#AT1, Alveolar Type 1 epithelial cell, pneumocyte
'AGER', 'RTKN2', 'CLIC5',

#AT2, Alveolar Type 2 epithelial cell
'SFTPC', 'SFTPA1', 'SFTPA2', 'WIF1', 'HHIP', 'CA2', 'ETV5' , 'WIF1', 'HHIP',

#Dividing
'MKI67','CDK1', 'TOP2A',

#Ciliated
'GSTA1', 'DTHD1', 'DCDC2B',

#Ciliated and Deuterosomal
'PIFO', 'FOXJ1' , 'CCDC78',

#Deuterosomal
'CDC20B', 'CDC20', 'FOXN4', 'CCNO',

#Secretory_Club & Secretory_Goblet
'CEACAM6', 'SCGB1A1',

#Secretory_Club
'SCGB3A2',

#Secretory_Goblet
'MUC5AC', 'TSPAN8','CYP2F1', 'CEACAM5', 'VSIG2', 'FUT6',

#SMG_Mucous, Sub-mucosal glands, mucous secreting cells
'BPIFB2', 'MUC5B', 'TFF3','TFF1',

#SMG_Serous, Sub-mucosal glands, serous secreting cells
'PRR4', 'LPO', 'PIP','S100A1', 'PRB3', 'C6orf58', 'PRB4', 'ODAM', 'PRH2',

#SMG_duct, Submucosal gland collecting duct columnar cells
'CLU', 'PROM1', 'RARRES1', 'CCL28', 'ALDH1A3', 'PI3', 'MIA','KRT23', 'KRT7',
'SCGB3A1', 'RARRES1', 'ZNHIT6', 'KRT7', 'WNT5B',

SMG Basal, Basal cells of the sub-mucosal glands
'PTK2B', 'MMP2', 'COL14A1', 'NUAK1',"G0S2",

#Basal and suprabasal
'KRT5', 'TP63', 'S100A2', 'KRT6A', 'TNS4',

#Basal airway epithelia
'MMP10', 'KRT14', 'DLK2', 'KRT15', 'COL17A1', 'LOXL4',

#Suprabasal
'LY6D', 'PLAT','SERPINB4',

#Ionocyte
'FOXI1', 'CFTR', 'ASCL3',

#Brush
'HEPACAM2', 'PLCG2', 'BIK',

#Neuroendocrine
'CHGA', 'CALCA', 'ASCL1', 'CHGB', 'GRP' ,'BEX1',

#Myoepithelial and Basal
"TP63", "KRT14", "KRT15", "KRT5", "KRT17", 'USP31',

#Myoepithelial and muscle
"ACTG2", 'ACTA2', 'TAGLN', 'CNN1',

#Myoepithelial
'LAMA1', 'PLD5', 'FHOD3',

Fibroblast markers

#Fibroblast
'VCAN', 'PDGFRA',

Alveolar fibroblast
'TCF21', 'ITGA8', 'FGFR4',

Adventitial fibroblast
'SFRP2', 'PI16','FBLN2', 'CD248', 'MFAP5',

#Immune recruiting fibroblast
'CXCL12',

#Fibroblast, T reticular Cells in lymph nodes & #Immune recruiting fibroblast
'CCL21', 'CCL19', 'GREM1',

#Follicular DC-s & #Immune recruiting fibroblast
'CXCL13', 'FDCSP',

#Myofibroblast
'ITGBL1', 'ASPN', 'TSPAN8', 'LMOD1', 'NALCN', 'ITGBL1', 'WISP2', 'F2R', 'WIF1', 'CCDC68', 'CHRM2',

Peribronchial Fibroblast:
"TMEM132C", "PAPPA", "COL13A1", "ATRNL1", "PLPPR4", "LHFPL3", "RGN", "CORIN" ,
'F13A1','LINC01436','FGF14', 'CHN1', 'PAPPA', 'PLCL1','NTRK3', 'SUGCT', 'PRDM6', 'ENOX1', 'ENTPD1', 'PDE3B', 'ATRNL1', 'COL15A1', 'PAG1',

#Perichondrial fibroblast
'KDR', 'FGFR2', 'TMTC1', 'COL12A1', 'CHI3L1', 'RUNX2', 'SCARA3', 'ZFHX4', 'GRID2', 'PCDH11X',

#Chondrocyte
'ACAN' ,'CHAD' ,'COL9A3','HAPLN1','COL2A1','CYTL1',

#Mesothelia
'MSLN', 'UPK3B', 'WT1', 'CALB2', 'HP', 'PRG4', 'ITLN1',

#Nerve-associated fibroblast (NAF)
'NGFR', 'TENM2','EBF2', 'THBS4',

#Endoneurial NAF
'ANGPTL7', 'APOD', 'AMD1', 'CDH19', 'TIAM1', 'USP54',

#Perineurial NAF
'PDZRN4', 'FGL2', 'SLC2A1', 'ITGA6', 'SORBS1','STXBP6','SLC22A3',

#Schwann
'PLP1' ,'MPZ','S100B',

#Myelinating Schwann
'GLDN','CDH7','DRP2','NFASC', 'NCMAP', 'MBP', 'PRX', 'MLIP',

#Non-myelinating Schwann:
'ADGRB3', 'ADGRL3', 'ARHGEF26','ADAM23','ARHGAP15','NRXN3', 'NRXN1', 'NCAM1', 'NCAM2', 'NLGN4X', 'NTM', 'NKAIN3', 'CADM2', 'CHL1', 'CADM1', 'CDH19','GRIK2', 'DOCK5' ,'COL21A1', 'CDH19', 'CADM4' , 'SCN7A', 'SORBS2', 'STARD13', 'SLC35F1', 'SOX6', 'SCN9A','SOX10', 'PPP2R2B', 'PCDH9', 'PTPRZ1', 'PRIMA1', 'PTPRJ', 'PDE1C','PLCE1', 'TENM3', 'TMOD2', 'TTYH1', 'FRMD4A', 'FRMD5', 'FAM129A', 'KIAA1217', 'KCNMB4','KHDRBS3', 'L1CAM','ERBB3', 'HAND2-AS1', 'LGI4', 'FIGN', 'ITGB4', 'IQGAP2', 'XKR4', 'IQGAP2', 'HAND2', 'ZSWIM6'

##Endothelial markers

#Endothelia
'PECAM1',

#Endothelia vascular
'AQP1', 'VWF',

#Endothelia lymphatic
'CCL21', 'PROX1', 'FLT4',

#Endothelia vascular capillary, Cap-g
'CA4', 'FCN3', 'SLC6A4', 'IL7R',

#Endothelia vascular capillary, Cap-a / Car4 / Aerocyte
'HPGD', 'EDNRB', 'IL1RL1', 'S100A3',

#Endothelia vascular arterial
'GJA5', 'DKK2', 'BMX',

#Endothelia vascular venous
'SELE', 'ACKR1', 'PLVAP',

#Endothelia vascular venous pulmonary
'CPE', 'DKK3', 'PDZRN4', 'EFEMP1', 'CDH11', "PTGS1", "MMRN1", "PKHD1L1", "HDAC9",

#Endothelia vascular venous systemic
'COL15A1', 'ZNF385D', 'EBF1', 'TSHZ2', "FLRT2", "OLFM1", "CPXM2", "TPD52L1",

Smooth muscle markers

##Smooth muscle
'TAGLN', 'ACTA2','TPM2', 'MYH11', 'CNN1',

#Smooth muscle (no pericytes)
'ACTG2', 'DES',

#Airway smooth muscle

'HPSE2','COL4A6','PRUNE2','NRP2','SEMA3E' , 'PCDH7', 'DOCK3', 'GPM6A','HS6ST3', "LGR6", 'PRUNE2', 'BCHE', #'SCARA3', 'SCUBE1',

#Smooth muscle systemic arteries
'SORBS2', 'SYNM', 'PHLDA2', 'NET1',

#Muscle smooth vascular pulmonary
'ELN', 'COL1A1', 'TGM2', 'TNNT2',

Pericyte
'RGS5', "ABCC9", 'CSPG4'

#Pericyte Parenchyma (CSPG4 negative)
'HIGD1B', 'COX4I2', 'PTN', 'LAMC3', 'MEST', 'KCNK3',

#Pericyte immune recruiting
'CCL19', 'CCL2', 'CXCL12', 'CCL21', 'FGF7', 'TNC', 'CLSTN2', 'CHSY3' ,'FABP4', 'STEAP4',

#Pericyte systemic
'SLC7A2', "FRMD3", "SLC38A11", "NEURL1B", "NCKAP5", "SLC38A11",

##Immune markers

Immune
'PTPRC',

##Lymphoid

#B cell
'CD19', 'CD79A', 'MS4A1','BANK1','LINC00926', 'BLK',

#B memory/ mature
'CD27', 'TNFRSF13B',

#B naive
'IGHD', 'FCER2', 'TCL1A',

#Proliferating cells, (Plasmablast if have plasma cell markers)
'MKI67', 'CDK1', 'TOP2A',

#Plasma cells
'MZB1', 'SDC1',

#B plasma IgG
'IGHG1', 'IGHG3', 'IGHG2',

#B plasma IgA
'IGHA1', 'IGHA2', 'CCR10',

#B plasma and DC plasmacytoid
'JCHAIN',

#DC plasmacytoid
'CLEC4C', 'PLD4', 'LILRA4', 'CXCR3', 'IL3RA',

T& NK

T cells
'CD3E','CD3G','CD3D',

CD4 T-cells
'CD4','CD40LG','CCR6','CXCR6',

CD4_TRM

'ITGA1','ITGAE','ZNF683','CCL4','CCL5','PFN1' , 'GZMA','GZMB',
#note CCL4, CCL5,PFN1, GZMA, GZMB are expressed in CD8 and NK, but for CD4 they are expressed mainly in the TRM population

CD4_naive/CM
'LTB','LEF1','CD28','KLF2','SELL',

CD8
'CD8A','CD8B',

CD8_EM/EMRA
'GZMH','KLRG1','PFN1','GZMA',

#CD8_TRM/EM
'GZMK','EOMES','DTHD1','CRTAM', 'LYST', 'TNIP3',

CD8_TRM
'GZMB','CCL4','CCL5','ITGA1','PDCD1','ITGAE' ,'ZNF683',

ILC
'AREG','SOX4','KIT','TNFRSF18',

MAIT
'KLRB1','IL7R','IFNGR1','SLC4A10','TRAV1-2','TRBV6-2','DUSP2',

NK_CD16hi
'FCER1G','GNLY','KLRF1','KIR2DL1','GZMB', 'FGFBP2', 'NKG7',

NKT, also T cell and NK cell markers already in the marker gene list
'S1PR1','IL32',

NK_CD11d
'ITGAD','CD247','KIR3DX1','ITGAX', 'KLRC3',

NK_CD56bright
'XCL1','CMC1','CD7','CCL3','NCR1','IL2RB','SRGN',

Tregs
'FOXP3','CCR4','CTLA4','IL2RA','TNFRSF4','TIGIT',

gdT
'KLRC2','TRDC','TRDV1','TRG-AS1','TRGC1','KIR2DL4',

#Erythrocyte
'HBA1', 'HBA2', 'HBD', 'HBB',

Myeloid
'AIF1','LYZ', 'COTL1',

#DC_1, (cDC1, conventional dendritic cell 1)
'CLEC9A', 'XCR1', 'C1orf54', 'WDFY4', 'LGALS2', 'DNASE1L3',

#DC_1 and DC activated
'IDO1',

#DC activated
'CCR7', 'BIRC3', 'RASSF4', 'TRAF1', 'EBI3',

#DC_2, (cDC2, conventional dendritic cell 2)
'CD1C', 'CLEC10A', 'FCGR2B', 'FCER1A',

#Monocyte
'CD300E', 'FCN1',

#Monocyte_CD14
'S100A12', 'EREG', 'CD14',

#Monocyte_CD16
'FCGR3A', 'LILRB2', 'LILRB1', 'MTSS1', 'FAM110A', 'LRRC25', 'CDKN1C', 'ABI3',

#Macrophage
'C1QA', 'C1QB', 'C1QC', 'APOE', 'APOC1',

#Macrophage alveolar, Macro_alv
'MARCO', 'MCEMP1', 'INHBA', 'TREM1', 'ABHD5', 'PPARG', 'RETN', 'CD5L', 'FABP4',

#Macrophage alveolar metallothioneins
'MT2A', 'MT1X', 'MT1G', 'MT1F', 'MT1H',

#Macrophage CHIT1
'SPP1', 'PLA2G7', 'FOLR2', 'SLC1A3', 'SDC2', 'CHIT1', 'OTOA',

#Macrophage interstitial
'CXCL10', 'CXCL9', "CXCL11",'GBP1', 'GBP5','GBP4', 'GBP2', 'PSTPIP2', 'SLAMF7', 'WARS','STAT1','GCH1', 'APOL3',

#Macrophage intravascular
'LILRB5','F13A1', 'STAB1', 'RNASE1', 'MAF', 'FOLR2', "LYVE1",

#Macrophage CCL
'CCL4', 'CCL20', 'CCL4L2', 'CXCL3', 'CXCL8', 'CCL20', 'IL1B', 'CXCL5', 'CXCL2',

Macro airway CX3CR1
'CX3CR1', 'RGS1', 'C3', 'PALD1', 'MEF2C', 'DOCK4','EPB41L2', 'ADAM28',
'SERPINB9', 'ST6GAL1', 'FCGBP', 'SRGAP1', 'NFATC2', 'IGSF21', 'BCL2',
'SFMBT2', 'ATP8B4', 'INPP5D', 'SLC4A7', 'SIGLEC8', 'SIGLEC10', 'PLD4', 'HAMP',

#Mast_cell
'TPSAB1', 'TPSB2', 'CPA3', 'HPGDS', 'MS4A2', 'SLC18A2', 'TPSD1', 'RGS13', 'HDC',

#Megakaryocyte
'TUBB1', 'ANK1', 'PF4', 'TUBB1', 'CMTM5','PCSK6', 'STON2', 'PRKAR2B', 'SYTL4', 'LTBP1',

#Platelets/megakaryocytes
'PF4', 'GP9',
