# Supplementary material for: A spatially resolved atlas of the human lung characterizes a gland-associated immune niche
Source: Nat Genet. 2022 Dec 21;55(1):66–77. doi: 10.1038/s41588-022-01243-4 (PMC9839452; doi:10.1038/s41588-022-01243-4)
Supplement: Supplementary file 2 — Reporting Summary [file 41588_2022_1243_MOESM2_ESM.pdf]

nature research

Corresponding author(s): Kerstin B Meyer
Sarah A Teichmann

Last updated by author(s): Sep 12, 2022

Reporting Summary

Nature Research wishes to improve the reproducibility of the work that we publish. This form provides structure for consistency and transparency in reporting. For further information on Nature Research policies, see our Editorial Policies and the Editorial Policy Checklist.

Statistics

For all statistical analyses, confirm that the following items are present in the figure legend, table legend, main text, or Methods section.

n/a	Confirmed	
☐	☒	The exact sample size (*n*) for each experimental group/condition, given as a discrete number and unit of measurement
☐	☒	A statement on whether measurements were taken from distinct samples or whether the same sample was measured repeatedly
☐	☒	The statistical test(s) used AND whether they are one- or two-sided<br>*Only common tests should be described solely by name; describe more complex techniques in the Methods section.*
☐	☒	A description of all covariates tested
☐	☒	A description of any assumptions or corrections, such as tests of normality and adjustment for multiple comparisons
☒	☐	A full description of the statistical parameters including central tendency (e.g. means) or other basic estimates (e.g. regression coefficient) AND variation (e.g. standard deviation) or associated estimates of uncertainty (e.g. confidence intervals)
☐	☒	For null hypothesis testing, the test statistic (e.g. *F*, *t*, *r*) with confidence intervals, effect sizes, degrees of freedom and *P* value noted<br>*Give P values as exact values whenever suitable.*
☒	☐	For Bayesian analysis, information on the choice of priors and Markov chain Monte Carlo settings
☒	☐	For hierarchical and complex designs, identification of the appropriate level for tests and full reporting of outcomes
☐	☒	Estimates of effect sizes (e.g. Cohen's *d*, Pearson's *r*), indicating how they were calculated

Our web collection on statistics for biologists contains articles on many of the points above.

Software and code

Policy information about availability of computer code

Data collection	For this project sequencing data was generated on Illumina NovaSeq machines and analysed using the open source software listed in the next section.
Data analysis	The following software and code was used, with version number where available.<br><br>Space Ranger v1.1.0<br>Cell Ranger v4.0.0<br>Cell2location v0.1<br>SoupX v1.0.0<br>Harmony v1.0<br>BBKNN v1.4.1<br>scVI-tools v0.9.0<br>scanpy v1.7.1<br>gProfileR e106_eg53_p16_65fcd97<br>CellChat v1.1.1<br>scVelo package v0.2.1<br>Azimuth v0.4.1<br>CellTypist v1.2.0<br>Scirpy v0.6.0<br>Milopy v0.0.999<br>Omero v5.14.1<br>Imaris v9.7.0

Custom code is available on our Github repository https://github.com/elo073/5loclung, DOI 10.5281/zenodo.7125810.

For manuscripts utilizing custom algorithms or software that are central to the research but not yet described in published literature, software must be made available to editors and reviewers. We strongly encourage code deposition in a community repository (e.g. GitHub). See the Nature Research guidelines for submitting code & software for further information.

Data

Policy information about availability of data

All manuscripts must include a data availability statement. This statement should provide the following information, where applicable:
- Accession codes, unique identifiers, or web links for publicly available datasets
- A list of figures that have associated raw data
- A description of any restrictions on data availability

All transcriptomic data generated as part of the study are publicly available. The processed scRNA-seq, snRNA-seq and Visium spatial transcriptomics data is available for browsing and download via our website www.lungcellatlas.org. The dataset (raw data and metadata) is available on the Human Cell Atlas Data Portal and on the European Nucleotide Archive (ENA) under accession number PRJEB52292 and BioStudies accession S-SUBS17. The Visium data is publicly available on ArrayExpress with the accession number E-MTAB-11640. Imaging data can be downloaded from European Bioinformatics Institute (EBI) BioImage Archive under accession number S-BIAD570. Additional data was accessed to support analysis and conclusions, which can be accessed through National Centre for Biotechnology Information Gene Expression Omnibus GSE136831, and GSE134174 and the Human Lung Cell Atlas integration which can be accessed through github https://github.com/LungCellAtlas/HLCA.

Field-specific reporting

Please select the one below that is the best fit for your research. If you are not sure, read the appropriate sections before making your selection.

☒ Life sciences ☐ Behavioural & social sciences ☐ Ecological, evolutionary & environmental sciences

For a reference copy of the document with all sections, see nature.com/documents/nr-reporting-summary-flat.pdf

Life sciences study design

All studies must disclose on these points even when the disclosure is negative.

Sample size	Samples were obtained from a total of 12 human organ donors (7 donors for scRNA-seq, 7 donors for snRNA-seq and 6 donors for Visium ST as in Supplementary tables 1-3).<br>Total number of single cell and single nuclei transcriptomes analysed: 193,108<br>No Sample size calculation was carried out as we are not comparing distinct phenotypes but describing the cell types present in normal healthy lung and airway. Due to the rarity of organ lung donor availability, samples were profiled in depth using multiple techniques rather than including many donors. The sample size in our study is sufficient as it is more than most other published single cell studies that have profiled healthy lung. All described populations were identified in at least three individuals.
Data exclusions	Thresholds common in single cell studies were applied to exclude poor quality cells (see methods for details). No individuals were excluded from the analysis. Five sections from the fresh frozen Visum ST experiment were excluded from Cell2location calculations due to poor quality.
Replication	All findings we report are supported by robust statistical analysis that is outlined in detail in the methods section of the paper. Any cell types we report were found in multiple individuals. For spatial transcriptomics analysis, a total of 20 sections were profiled however 5 were excluded due to low quality as stated in the methods. Cell type mapping by Visium spatial transcriptomics was reproducible in at least 3 sections.<br><br>When validating cell types by RNAscope, a minimum of 2 sections from 2 donors were stained and representative sections shown. For multiplex IHC experiments, 3 donor airway samples (both bronchi and trachea) were stained with at least 1 section per location per donor and representative sections shown. All replicate experiments were successful and reflect the results reported in the study.
Randomization	Randomisation was not applicable in the study. Patients that were available within organ donor programme were analysed immediately as fresh samples. The patients for frozen samples were pooled and analysed as soon as 5 patients were available. Sections for spatial methods were used specifically after screening for relevant structures on the sections.
Blinding	Blinding was not applicable to this study because all of our donors were healthy lung organ donors.

Reporting for specific materials, systems and methods

We require information from authors about some types of materials, experimental systems and methods used in many studies. Here, indicate whether each material, system or method listed is relevant to your study. If you are not sure if a list item applies to your research, read the appropriate section before selecting a response.

Materials & experimental systems

n/a	Involved in the study
☐	☒ Antibodies
☒	☐ Eukaryotic cell lines
☒	☐ Palaeontology and archaeology
☐	☒ Animals and other organisms
☐	☒ Human research participants
☒	☐ Clinical data
☒	☐ Dual use research of concern

Methods

n/a	Involved in the study
☒	☐ ChIP-seq
☒	☐ Flow cytometry
☒	☐ MRI-based neuroimaging

Antibodies

Antibodies used

Antibody details (supplier, catalog number, conjugate, clone name, cycle position for multiplexing and dilution) are given in Supplementary Table 6.

Validation

All antibodies are commercially available and validated, and we provide links to available technical datasheet below:
Hoechst 33258: https://biotium.com/wp-content/uploads/2016/12/PI-40044-40045.pdf
CD3: https://www.biolegend.com/fr-ch/products/alexa-fluor-488-anti-human-cd3-antibody-2726?pdf=true&displayInline=true&leftRightMargin=15&topBottomMargin=15&filename=Alexa%20Fluor%C2%AE%20488%20anti-human%20CD3%20Antibody.pdf
CD31: https://www.thermofisher.com/order/genome-database/dataSheetPdf?producttype=antibody&productsubtype=antibody_primary&productId=12-0319-42&version=224
HLA-DR: https://www.abcam.com/alexa-fluor-647-hla-dr-antibody-tal-1b5-ab223907.pdf
IgD: https://www.biolegend.com/en-us/products/alexa-fluor-488-anti-human-igd-antibody-7758?pdf=true&displayInline=true&leftRightMargin=15&topBottomMargin=15&filename=Alexa%20Fluor%C2%AE%20488%20anti-human%20IgD%20Antibody.pdf
CD4: https://www.biolegend.com/it-it/products/alexa-fluor-647-anti-human-cd4-antibody-2728?pdf=true&displayInline=true&leftRightMargin=15&topBottomMargin=15&filename=Alexa%20Fluor%C2%AE%20647%20anti-human%20CD4%20Antibody.pdf
IgA2: https://resources.southernbiotech.com/techbul/9140.pdf
EpCAM: https://www.thermofisher.com/order/genome-database/dataSheetPdf?producttype=antibody&productsubtype=antibody_primary&productId=50-9326-42&version=224
Phalloidin: https://www.biolegend.com/en-us/products/flash-phalloidin-green-488-13950?pdf=true&displayInline=true&leftRightMargin=15&topBottomMargin=15&filename=Flash%20Phalloidin%E2%84%A2%20Green%20488.pdf
CD45: https://www.biolegend.com/en-us/products/apc-anti-human-cd45-antibody-705?pdf=true&displayInline=true&leftRightMargin=15&topBottomMargin=15&filename=APC%20anti-human%20CD45%20Antibody.pdf
CD45RO: https://www.biolegend.com/en-us/products/alexa-fluor-488-anti-human-cd45ro-antibody-3340?pdf=true&displayInline=true&leftRightMargin=15&topBottomMargin=15&filename=Alexa%20Fluor%C2%AE%20488%20anti-human%20CD45RO%20Antibody.pdf
CD45RA:
https://www.biolegend.com/en-us/products/pe-anti-human-cd45ra-antibody-687?pdf=true&displayInline=true&leftRightMargin=15&topBottomMargin=15&filename=PE%20anti-human%20CD45RA%20Antibody.pdf
CCL28: https://www.atlasantibodies.com/api/print_datasheet/HPA077434.pdf
anti-rabbit IgG AF647: https://www.abcam.com/goat-rabbit-igg-hl-alexa-fluor-647-ab150079.html (link to datasheet download, antibody has 189 references as of 12 September 2022)
anti-mouse IgA PE: https://www.thermofisher.com/order/genome-database/dataSheetPdf?producttype=antibody&productsubtype=antibody_primary&productId=12-5994-81&version=251
Streptavidin PE: https://www.biolegend.com/it-it/products/pe-streptavidin-1475?pdf=true&displayInline=true&leftRightMargin=15&topBottomMargin=15&filename=PE%20Streptavidin.pdf&v=20220609101609

Animals and other organisms

Policy information about studies involving animals; ARRIVE guidelines recommended for reporting animal research

Laboratory animals

Wild-type C57/BL6 mouse samples were obtained from Kindai University, Japan (courtesy of Prof. Takashi Nakayama) and Charles River, USA (AMSbio). Male (colon samples) and female (all other samples) mice were and used at 8–10 weeks old. Mice from Kindai University were housed in specific pathogen free conditions with 12 hour light/dark cycle (light on from 7am to 7pm) at ambient temperature 22±1°C and 55±10% humidity. All animal experiments for mice obtained from Kindai University were approved by the Centre of Animal Experiments at Kindai University. Mice from Charles River were housed in specific pathogen free conditions with 12 hour light/dark cycle at ambient temperature 21±2°C and 30-70% humidity. Mouse tissue from Charles River was purchased from a certified animal supplier through AMSbio, with an internal ethical approval process for broadly defined research use.

Wild animals

No wild animals were used.

Field-collected samples

No field-collected samples were used.

Ethics oversight

Identify the organization(s) that approved or provided guidance on the study protocol, OR state that no ethical approval or guidance

Ethics oversight	*was required and explain why not.*

Note that full information on the approval of the study protocol must also be provided in the manuscript.

Human research participants

Policy information about studies involving human research participants

Population characteristics	All participants were deceased organ donors without prior history of lung disease. Patient characteristics such as age, BMI and sex are given in the Supplementary Table 1.
Recruitment	Samples from deceased organ donors were provided by the Cambridge Biorepository for Translational Medicine, and individual donors were selected on the basis of organ availability.
Ethics oversight	Samples were obtained from deceased transplant organ donors by the Cambridge Biorepository for Translational Medicine (CBTM) with informed consent from the donor families and approval from the NRES Committee of East of England – Cambridge South (15/EE/0152). This consent includes generation of open-access genetic sequencing data and publication in open access journals in line with Wellcome Trust policy. CBTM operates in accordance with UK Human Tissue Authority guidelines.

Note that full information on the approval of the study protocol must also be provided in the manuscript.
